# Advancements in Artificial Intelligence for Enhanced Diagnosis and Prognosis in Cardiac CT: a Comprehensive Review and Future Perspectives

**DOI:** 10.1007/s11886-026-02443-8

**Published:** 2026-11-05

**Authors:** Fatemeh Adabifirouzjaei, Matthew Budoff, Mo Nomeli

**Affiliations:** 1 The Division of Cardiology, University of California at Riverside, Riverside, 92521 USA; 2 Lindquist Research Institute, Harbor-UCLA Medical Center, Los Angeles, USA; 3 University of Maryland, College Park, USA

**Keywords:** Cardiac Computed Tomography, Artificial Intelligence, Coronary Artery Disease, Coronary Artery Calcium Scoring

## Abstract

**Purpose of Review:**

Cardiovascular disease, with coronary artery disease (CAD) as its main culprit, remains a leading cause of death worldwide. Traditional diagnostic methods that focus on the degree of arterial narrowing (percent diameter stenosis, or %DS) can miss early signs of disease or the specific plaque features that indicate high risk. The purpose of this review is to examine how artificial intelligence (AI) can enhance the diagnostic and prognostic capabilities of cardiac computed tomography (CCT), and to address where AI can most meaningfully improve diagnostic accuracy, workflow efficiency, and patient care.

**Recent Findings:**

AI is transforming cardiac CT across the entire imaging pathway. By applying AI algorithms, recent studies have refined the assessment of coronary artery calcium (CAC), improved plaque characterization, and detected subtle lesions that might evade traditional detection methods. AI further enables non-invasive assessment of ischemia (reduced blood flow), aids in the evaluation of myocardial infarction, analyzes adipose tissue surrounding the heart, improves image quality, and provides valuable prognostic insights for risk stratification.

**Summary:**

This comprehensive review explores the transformative potential of AI in cardiac CT, highlighting its role in accurate diagnosis, precise risk stratification, and personalized management of cardiovascular disease. Through collaboration between AI technology and clinical expertise, we envision a future where cardiac CT imaging is more powerful, efficient, and patient-centered, ultimately leading to improved outcomes in cardiovascular medicine. Continued standardization, external validation, and clinician education will be essential to realize this impact in routine practice.

## Introduction

Cardiovascular disease, particularly coronary artery disease (CAD), is a leading global health concern, primarily driven by atherosclerosis, the underlying cause of most heart attacks and other cardiac events. Despite traditional diagnostic methods focusing on the degree of arterial narrowing (percent diameter stenosis, or %DS), a significant portion of dangerous lesions often go undetected due to their lack of significant obstruction [1]. To address this critical gap, the evaluation of plaque characteristics, including high-risk plaque (HRP), plaque burden, and plaque composition, has become paramount in identifying individuals at risk beyond simple stenosis measurements [2].

Recent research has highlighted the importance of low-attenuation plaque burden as a strong predictor of future heart attacks, further emphasizing the need for advanced imaging beyond traditional stenosis assessment [3, 4].

Coronary computed tomography angiography (CCTA) has emerged as a powerful tool in this regard, providing detailed insights into coronary circulation, luminal stenosis, and atherosclerotic plaque morphology [5]. Beyond anatomical features, the functional significance of coronary plaques, assessed using fractional flow reserve (FFR), has gained recognition in guiding treatment decisions [6]. Additionally, the emerging role of pericoronary adipose tissue (PCAT) as a cardiovascular risk marker suggests its potential integration into routine CCTA imaging for personalized risk assessment [7].

In this rapidly evolving landscape, artificial intelligence (AI) has emerged as a game-changer, promising to automate and enhance various aspects of cardiac CT imaging. By leveraging AI algorithms, we can extract and analyze vast amounts of data from medical images and electronic health records, enabling personalized risk assessment and prediction of future cardiac events. This comprehensive review explores the latest advancements in AI-driven approaches for diagnosing coronary calcium scoring, characterizing coronary plaques, and predicting outcomes in coronary computed tomography (CCT) imaging. We will delve into the methodologies, challenges, and exciting future directions of this transformative technology in cardiovascular medicine.

### Basic Principals

#### Machine Learning

Machine learning (ML), in the context of cardiac CT, empowers algorithms to learn from data, mirroring the human capacity for adaptation and improvement. By analyzing large datasets of cardiac CT scans, ML models identify intricate patterns and relationships, enabling them to make predictions or inferences on new, unseen data. This eliminates the need for explicit programming for each specific diagnostic task [8].

ML encompasses three primary paradigms: (Table 1).

- Supervised Learning: Algorithms learn from labeled data (e.g., CT scans with expert annotations) to classify or predict outcomes (e.g., presence of coronary artery disease).
- Unsupervised Learning: Algorithms uncover hidden structures and relationships within unlabeled data, potentially revealing novel biomarkers or disease subtypes.
- Reinforcement Learning: Algorithms learn optimal strategies through trial and error, guided by feedback from their environment. This approach shows promise in optimizing treatment plans or guiding interventions [9].

**Table 1:** The Summary of Key Machine Learning Concepts

Concept	Description	Application in Cardiac CT
Supervised Learning	Learning from labeled data (e.g., CT scans with expert annotations) to predict outcomes or classify data points	Predicting the presence and severity of coronary artery stenosis, classifying plaque types, detecting high-risk plaque features
Unsupervised Learning	Discovering hidden patterns or structures in unlabeled data	Identifying novel subtypes of coronary artery disease, clustering patients based on plaque characteristics, anomaly detection
Reinforcement Learning	Learning optimal strategies through trial and error, guided by feedback from the environment	Optimizing radiation dose protocols for cardiac CT scans, potentially guiding personalized treatment decisions
Deep Learning (DL)	A subset of ML that uses artificial neural networks with multiple layers to learn complex representations	Image segmentation (identifying heart chambers, vessels), automatic plaque detection and quantification, predicting myocardial perfusion
DL-Convolutional Neural Networks (CNNs)	Specialized neural networks designed for image analysis	Extracting features from cardiac CT images (e.g., texture, shape) to aid in diagnosis and prognosis
DL-Recurrent Neural Networks (RNNs)	Neural networks designed for processing sequential data (e.g., time series of cardiac function)	Analyzing cardiac motion patterns, predicting future cardiac events based on longitudinal data

Deep Learning (DL), a subset of ML, employs multi-layered artificial neural networks to excel at complex tasks like image segmentation and feature extraction [10]. Within DL, several architectures have proven particularly valuable in cardiac CT:

- Convolutional Neural Networks (CNNs): These have revolutionized cardiac CT analysis by automatically identifying anatomical structures and disease markers within images [11].
- Recurrent Neural Networks (RNNs): These are specialized for sequential data, making them suitable for analyzing cardiac motion patterns over time or predicting future cardiac events based on longitudinal CT scans [12].

The performance of DL models is contingent on factors like network architecture, the quality and quantity of training data, and the choice of optimization algorithms. Please refer to Table 1 for a summary of key ML concepts used in this article.

#### Radiomics

Radiomics, an innovative AI-driven approach, transcends traditional visual assessments by extracting quantitative features from medical images. In cardiac CT, particularly CCTA and non-contrast cardiac CT, radiomics analysis goes beyond assessing stenosis and calcification. It delves into the intricate texture and spatial arrangement of Hounsfield Units (HU) or signal intensities within images, enabling comprehensive characterization of coronary plaques, pericoronary adipose tissue (PCAT), and myocardium. By unveiling these hidden patterns, radiomics promises to revolutionize cardiac imaging, paving the way for personalized, data-driven diagnostics and prognostics [13, 14].

### AI in CT Diagnostics

**Fig. 1:**
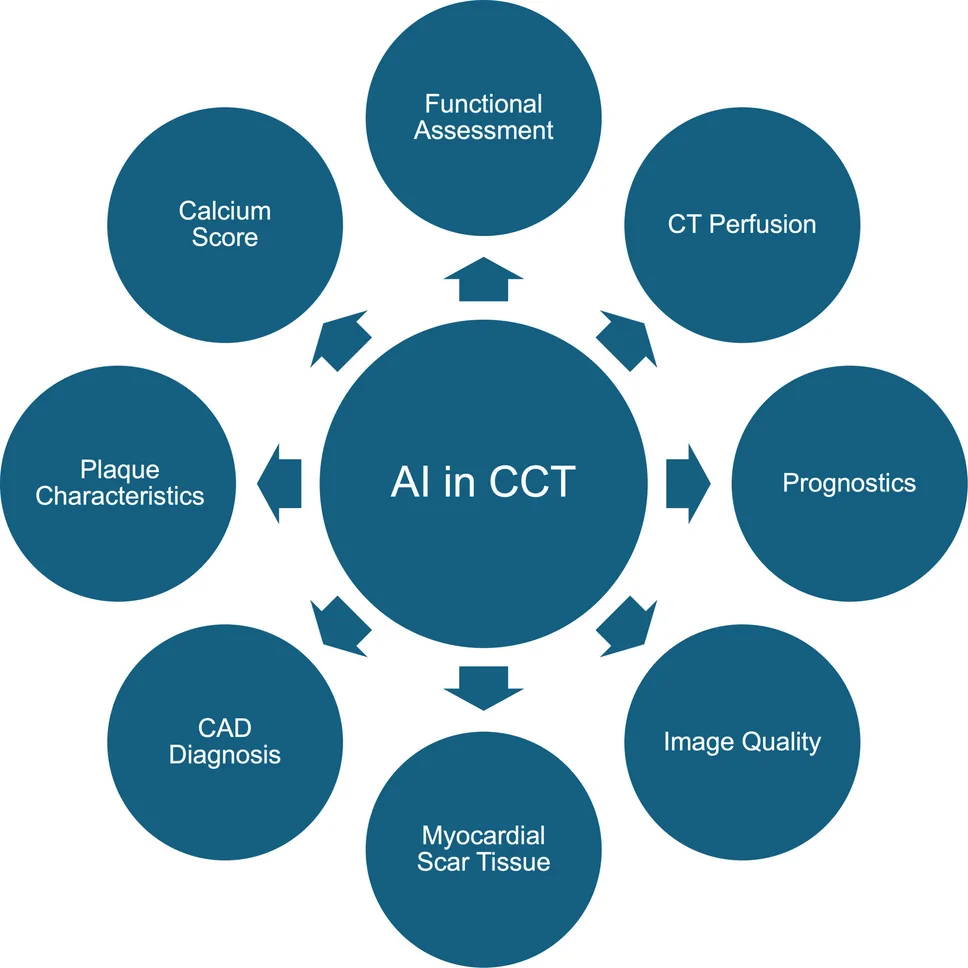
Principal applications of artificial intelligence (AI) in cardiac CT, including calcium scoring, plaque characterization, CAD diagnosis, ischemia and perfusion assessment, myocardial scar evaluation, prognostication, and image quality

**Fig. 2:**
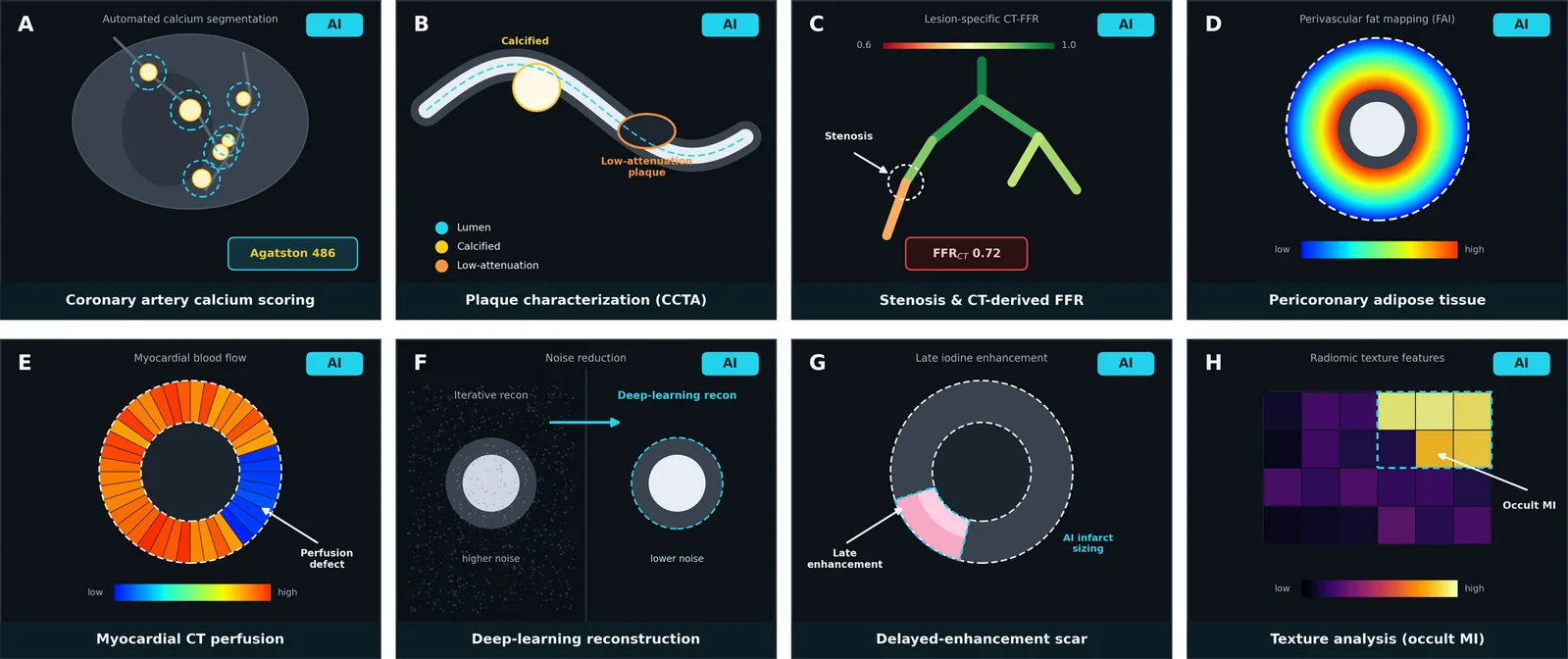
Representative applications of artificial intelligence (AI) across the cardiac CT examination. Schematic illustrations show where AI contributes to image analysis. (**A**) Automated coronary artery calcium scoring, with AI segmentation of calcified foci for Agatston quantification. (**B**) Coronary plaque characterization on CCTA, differentiating the vessel lumen, calcified plaque, and low-attenuation (lipid-rich) plaque. (**C**) Stenosis detection with CT-derived fractional flow reserve (CT-FFR), showing lesion-specific, colour-coded FFR values along the coronary tree. (**D**) Pericoronary adipose tissue assessment, with perivascular fat mapping and the fat attenuation index (FAI). (**E**) Myocardial CT perfusion, with AI delineation of a regional perfusion defect on a short-axis map of myocardial blood flow. (**F**) Deep-learning image reconstruction, yielding lower image noise than iterative reconstruction. (**G**) Delayed-enhancement CT of myocardial scar, with AI contouring and quantification of the region of late iodine enhancement (infarct). (**H**) Radiomic texture analysis, in which quantitative texture features identify occult myocardial infarction not evident on visual inspection. Dashed contours and colour overlays denote AI-derived segmentations and measurements. The panels are schematic representations created for illustration and do not depict specific patient data

Recent advancements in artificial intelligence have significantly broadened the scope of CCT, offering innovative tools for detailed assessment and diagnosis. From evaluating calcium scores to predicting prognostics and enhancing image quality, AI-integrated CCT has become pivotal in non-invasive cardiology (Fig. 1; representative examples are shown in Fig. 2).

#### Calcium Scoring

The coronary artery calcium (CAC) score, calculated from non-contrast CT scans, serves as a simple yet powerful tool for assessing atherosclerotic disease. This score, which measures the amount of calcium buildup in the coronary arteries, is a well-established independent predictor of major adverse cardiovascular events. Traditionally, this has been assessed through semi-automatic segmentation of calcified plaques with a density greater than 130 HU on axial CT images [15].

In recent years, AI has been transforming CAC scoring. DL algorithms have demonstrated remarkable ability to automatically and accurately calculate CAC scores across various CT protocols, even outperforming traditional methods in some cases. Notably, research by Van Vezlen et al. highlighted the robustness of DL in this domain, with further improvements achievable through tailored training data [16]. In another study, Martin et al. found that DL-based software could accurately calculate CAC scores in over 500patients, potentially streamlining clinical workflows while maintaining accuracy [17].

Beyond mere calculation, AI is also refining the way we measure CAC [18]. Studies comparing different reconstruction techniques, such as PureCalcium reconstructions versus virtual non-contrast (VNC) and true non-contrast (TNC) acquisitions, have revealed the superior accuracy of AI-enhanced methods [19]. Furthermore, by automating CAC quantification in CCTA scans, AI could potentially eliminate the need for dedicated CAC scoring scans, reducing radiation exposure for patients [11].

#### Plaque Characteristics

AI, combined with CCTA, has revolutionized our understanding of coronary plaque characteristics and their impact on CAD prognosis. CCTA provides unparalleled visualization of coronary arteries, allowing us to identify and characterize both the degree of stenosis (narrowing) and the composition of the plaque itself, which are critical for tailoring management strategies in patients with coronary artery disease [2].

CCTA allows for the assessment of not only the morphology (shape and size) but also the function of coronary plaques. This includes differentiating between calcified and non-calcified plaques and identifying high-risk plaque features like low attenuation plaque, positive remodeling, and spotty calcifications, which are often associated with culprit lesions in acute coronary syndrome (ACS) and poor outcomes [20–22].

Recent AI advancements have propelled us into an era of precision medicine for cardiovascular care. A multi-task recurrent convolutional neural network (CNN) applied to multi-planar reformatted (MPR) images of the coronary arteries has demonstrated promising results in automatic detection and classification of coronary artery plaque and stenosis [23]. By utilizing a 3D CNN for feature extraction along the coronary artery and aggregating those features with a recurrent neural network, this method can simultaneously detect and characterize plaque type (no plaque, non-calcified, mixed, calcified) and stenosis severity (no stenosis, non-significant < 50% luminal narrowing, significant > 50% luminal narrowing), achieving accuracies of 0.77 and 0.80 respectively. This innovation may enable automated triage of patients, identifying those without coronary plaque and those with plaque and stenosis who require further cardiovascular workup.

Automated analysis methods and software designed to specifically assess low-attenuation plaque (LAP) have also shown significant promise [23]. For example, Matsumoto et al. demonstrated that AI algorithms can greatly improve the correlation between LAP and the presence and size of lipid-rich components (LRC), the dangerous parts of plaques prone to rupture. This could lead to more accurate risk stratification and targeted interventions for patients [24].

Deep learning (DL) systems have also proven their worth in accurately quantifying plaque volume and stenosis severity, even matching the performance of expert readers and intravascular ultrasound [25]. Additionally, ML algorithms utilizing CT-FFR and ΔCT-FFR values show promise in predicting proximal plaque formation, potentially allowing for early intervention and prevention of adverse events like heart attacks [26]. By enabling early intervention and prevention of adverse events such as myocardial infarction, these innovative approaches underscore the transformative impact of AI in cardiovascular risk assessment and management.

#### Diagnosis of CAD

AI is ushering in a new era for CAD diagnosis with CCTA. AI algorithms have shown remarkable potential in automated lesion detection and diagnosis, improving both sensitivity and consistency between different observers [27, 28]. This is particularly evident in cloud-based AI software, which has demonstrated high accuracy in detecting coronary artery stenosis, leading to faster and more precise evaluation of high-grade stenoses [29, 30].

These AI-driven tools extend beyond simple lesion detection to encompass comprehensive CAD evaluation, including plaque characterization and stenosis severity assessment [23, 31, 32].Deep learning networks have been successfully used for automated plaque detection, quantification, and classification, achieving high accuracy rates and paving the way for automated triage and diagnosis [33, 34].

AI also offers a promising avenue for identifying functionally significant stenoses by analyzing the left ventricular myocardium in conjunction with CCTA. This could help optimize patient management and reduce unnecessary invasive procedures [35–38].

As AI continues to evolve, ongoing standardization and validation efforts will be crucial for its seamless integration into clinical practice, ultimately transforming the way we diagnose and manage CAD through CCTA.

### AI in CT-Guided Ischemia Assessment

#### Functional Assessment

The use of AI in CT-FFR assessment has made significant strides. Combining CCTA with DL analysis of the left ventricular myocardium (LVM) has proven to be more accurate in identifying functionally significant coronary artery stenosis, potentially reducing the need for invasive procedures like invasive FFR measurements [39, 40].

ML integration in large-scale studies has also improved the prediction of ischemia risk. By considering multiple factors beyond just stenosis and plaque volume, AI algorithms have shown increased discriminatory power in identifying lesion-specific ischemia compared to traditional CCTA stenosis grading alone [41, 42].

Novel computational fluid dynamics-based FFR algorithms for CCTA have also demonstrated impressive performance, particularly in detecting ischemia in intermediate lesions, the "gray zone" where traditional methods often struggle. These algorithms have the potential to accurately diagnose ischemia in cases where stenosis assessment alone might be inconclusive [43].

Recent research has shown promising results with ML-based CT-FFR in diagnosing ischemia-causing lesions. It has outperformed traditional measures like diameter stenosis and even rivals the combination of diameter stenosis and myocardium jeopardy index (MJI), achieving an accuracy of 82% and an area under the curve (AUC) of 0.864 [44]. Furthermore, combining ML-based CT-FFR with additional parameters like Duke jeopardy score (DJS) and minimal lumen diameter (MLD) allows for precise non-invasive differentiation between flow-limiting and non-flow-limiting lesions, enhancing diagnostic efficiency [45].

Importantly, new ML algorithms for CT-FFR computation offer comparable accuracy to established computational fluid dynamics (CFD)-based methods, but with the advantage of shorter processing times. This means faster and more efficient diagnosis of obstructive CAD without the need for invasive procedures [39, 40, 46–48].

Research has shown that a combination of ML techniques, including radiomics and DL, can perform on par with simulated FFR measurements. Notably, a fusion of these methods achieved the highest predictive accuracy for identifying the need for revascularization in specific coronary artery plaque segments, potentially offering a faster alternative to invasive testing [42, 49, 50].

ML-based CT-FFR has also demonstrated superior diagnostic accuracy compared to conventional CCTA alone, irrespective of the location of stenosis within the coronary arteries [51]. This suggests its potential for identifying hemodynamically significant stenoses regardless of their anatomical position. In addition, quantitative analysis of CCTA plaque burden alongside AI techniques has further enhanced the accuracy of determining hemodynamic significance, particularly in intermediate coronary lesions [36].

On-site CT-FFR, leveraging ML algorithms, has shown strong correlations with invasive reference standards like Instantaneous Wave-Free Ratio (iFR®) [52]. This points to the feasibility of non-invasive hemodynamic assessment of CAD with high sensitivity and specificity, potentially transforming the way we assess and manage coronary artery disease.

#### CT Perfusion

AI is also transforming CT perfusion (CTP) imaging, particularly in myocardial perfusion assessments. A recent study introduced a cutting-edge DL-based image registration technique specifically designed for quantitative myocardial perfusion CT exams. This innovation effectively addresses challenges like low image quality, dynamic contrast changes, and misalignment due to cardiac stress and motion, resulting in significant improvements in accuracy and efficiency [53].

Furthermore, the integration of resting myocardial CTP analysis with CCTA, using ML methods, has shown great promise. By incorporating this additional information, the accuracy in identifying significant ischemia is significantly enhanced compared to relying on coronary stenosis alone [54]. This comprehensive approach, powered by AI, offers clinicians a more complete picture of cardiac function and pathology, ultimately leading to better-informed treatment decisions and improved patient outcomes.

### Myocardial Infarction and Scar Tissue

Recent research has highlighted the exciting potential of AI in evaluating myocardial scar tissue using cardiac CT imaging. By applying ML to analyze radiomic features—quantitative measures extracted from medical images—we can assess delayed enhancement CT scans and pinpoint scarred myocardium segments with accuracy similar to manual reading by experts [55].

Interestingly, texture analysis, a technique that examines the visual patterns within images, has been shown to be superior to subjective visual diagnosis in detecting myocardial infarction (MI) [56]. In fact, iterative reconstruction techniques can significantly improve the quality of images used for this analysis. Notably, a study by Mannil et al. demonstrated that texture analysis and ML could even detect MI cases that were not visually apparent to radiologists, showcasing the potential of AI to enhance diagnostic accuracy [57].

These findings collectively emphasize the transformative power of AI in refining cardiac CT imaging. The integration of AI-based methods offers the promise of more precise and efficient assessment of myocardial scar tissue, ultimately leading to better patient care and outcomes.

### Prognostication and Risk Stratification

Recent advances in applying AI to cardiovascular disease prediction have shown promising results. One example is a machine learning-based risk calculator that outperforms traditional guidelines by recommending statins to fewer individuals while still effectively identifying those at risk for cardiovascular events [58].

The integration of AI and radiomics into cardiac CT is transforming how we assess cardiovascular risk. Numerous studies have showcased the potential of AI models to enhance risk stratification and predict adverse outcomes like MI and even mortality [59–62]. For example, semi-automated plaque quantification provides additional prognostic information beyond clinical risk factors and traditional CT reading, further refining our ability to assess risk in patients undergoing CCTA [22].

Large-scale studies like the ADVANCE registry and CONFIRM registry have demonstrated the effectiveness of ML models in predicting major adverse cardiac events (MACE) and mortality [25, 63, 64]. These models leverage quantitative CT measurements, clinical risk factors, and biomarkers to create more accurate long-term predictions of cardiac events [25]. In fact, AI-driven analysis of CCTA data has outperformed conventional scores in identifying patients at risk for future cardiovascular events and predicting mortality [65, 66].

#### Epicardial and Perivascular Adipose Tissue

The volume and density of epicardial adipose tissue (EAT) and pericoronary adipose tissue (PCAT) are emerging as valuable biomarkers in cardiovascular risk assessment. EAT, which surrounds the heart, has been linked to issues like inflammation and triglyceride storage problems, while PCAT, located near the coronary arteries, may be a more specific indicator of coronary inflammation and CAD [67, 68].

AI applications in cardiac CT have revolutionized the assessment of EAT and PCAT [7]. Using CNNs, AI algorithms can accurately segment and measure these adipose tissues from CT images. This allows for large-scale studies of cardiometabolic risk markers, as demonstrated by high segmentation accuracy in studies like SCAPIS [69]. Importantly, AI is revealing complex relationships between EAT/PCAT characteristics and cardiovascular health. For example, lower EAT density and increased EAT volume have been associated with coronary calcification, elevated markers of plaque inflammation, and MACE [70].

AI-based quantification of EAT and PCAT volumes from cardiac CT scans has proven valuable in predicting CAD, atrial fibrillation, myocardial infarction, stroke, and mortality. In fact, PCAT models derived from CCTA show superior performance in predicting MACE, especially when combined with clinical factors [71–73]. Research has also found that EAT volume, combined with CCTA plaque measures and CT-FFR, can effectively predict ischemic CAD [74]. Furthermore, incorporating radiomic features of PCAT into coronary CCTA improves the identification of hemodynamically significant coronary artery stenosis compared to CCTA alone [75].

These advancements in AI-powered analysis of EAT and PCAT hold immense promise for more precise risk assessment and personalized treatment plans in cardiovascular medicine.

#### Radiomics

The combination of AI and radiomics is revolutionizing cardiac CT, enhancing diagnostic accuracy and predictive modeling in cardiovascular medicine. Radiomics analysis has been shown to improve the diagnostic performance of CCTA in identifying myocardial ischemia and predicting MACE [13, 76]. By combining AI with radiomics, we can achieve more accurate risk assessment and non-invasive evaluation of cardiac events.

One example is the work of Denzinger et al., who introduced ML approaches, including radiomics and DL, for assessing coronary artery plaque segments in CCTA scans [77]. These methods performed comparably to simulated FFR measurements, offering a potential pathway for fully automated risk assessment without time-consuming manual intervention [78].

Moreover, radiomics analysis goes beyond plaque characterization. It has also been applied to PCAT analysis, improving the prediction of future acute coronary syndrome (ACS) within three years [79]. Radiomic signatures of PCAT derived from CCTA outperform traditional plaque characteristics in predicting MACE, highlighting their potential clinical benefit. AI-driven models further enhance this approach by automating the extraction of quantitative features from CCTA images, allowing for effective plaque classification and precise phenotyping of culprit and non-culprit lesions in MI and stable CAD [78, 80].

The integration of AI and radiomics in cardiac CT holds immense potential for refining risk stratification, guiding clinical decision-making, and ultimately improving patient outcomes [81].

### Image Quality

Deep learning-based image restoration (DLR) is emerging as a powerful tool in coronary CT angiography (CCTA). Compared to traditional reconstruction methods like hybrid iterative reconstruction (IR) or filtered back projection (FBP), DLR consistently delivers superior image quality [82]. This is evident in multiple studies, where DLR images consistently exhibit lower noise, enhanced contrast-to-noise ratio (CNR), and overall improved clarity [83].

The advantages of DLR are especially pronounced in cases involving calcification, a common challenge in assessing the severity of arterial narrowing. By enhancing image quality in these scenarios, DLR not only improves diagnostic accuracy but also opens the door to potentially reducing radiation exposure, a crucial consideration in medical imaging [84].

The superiority of DLR is not just measurable; it's also visually apparent. Studies have shown that DLR images receive higher subjective scores from experts compared to traditional reconstructions, further confirming its benefits.

With its ability to dramatically improve image quality, DLR could revolutionize coronary CTA. We envision a future where noninvasive imaging combined with AI-powered analysis streamlines the diagnosis of vascular diseases, leading to more accurate results and ultimately better outcomes for patients. The evidence strongly suggests that DLR is poised to redefine the standards of image quality in cardiovascular imaging, ushering in a new era of precision and effectiveness.

## Future Perspective

The future of cardiac CT angiography (CCTA) is incredibly exciting, with artificial intelligence (AI) poised to transform its capabilities, particularly through the advancement of deep learning-based image reconstruction (DLR). Recent research has demonstrated DLR's remarkable ability to significantly reduce image noise in CCTA compared to conventional methods like adaptive statistical iterative reconstruction-Veo (ASiR-V), all while maintaining diagnostic accuracy [85]. This breakthrough has the potential to not only enhance image quality but also revolutionize various aspects of cardiovascular imaging, from improving prognostic accuracy to streamlining the delivery of care [86].

While AI promises to revolutionize cardiac CT, it's important to note that its role will likely complement, rather than replace, the expertise of human physicians. Clinicians will remain essential in validating AI-driven diagnoses, ensuring the accuracy and reliability of results. AI-based software in CCTA has already shown promise in enhancing the evaluation of high-risk plaque and plaque burden, but ongoing research is needed to address the nuances of certain conditions like coronary anomalies, myocardial bridging, and imaging artifacts [87]. Collaboration between AI technology and clinical expertise will be key to maximizing the benefits of AI for optimal patient care [9].

Together, AI and human expertise hold the potential to usher in a new era of precision and efficiency in cardiovascular imaging, leading to more accurate diagnoses, personalized treatment plans, and ultimately, improved outcomes for patients.

## Challenges

The path to integrating AI into cardiac CT isn't without its challenges. While AI shows great promise in improving diagnoses and recommending helpful tests, several obstacles remain. A key challenge is the absence of clear-cut gold standards for diagnosis, making it difficult to definitively assess the accuracy of AI algorithms. Additionally, electronic health record (EHR) data often requires extensive preprocessing due to its unstructured nature, making it difficult to incorporate into AI models seamlessly.

Understanding how cardiologists will interact with AI systems in their daily workflows is another crucial aspect. We need more randomized controlled trials (RCTs) to evaluate the true clinical impact of AI-assisted care and determine how it can best be integrated into existing practices.

When it comes to using AI for cardiovascular risk assessment, there are additional hurdles. ML models rely heavily on data availability and quality, which can be problematic for rare diseases or unusual presentations where data is scarce. Standardizing image acquisition protocols across institutions is vital for effective data integration. Moreover, the computational costs associated with handling large datasets can limit widespread access to AI, although ongoing advancements in hardware and software solutions are gradually addressing this issue.

Ensuring that AI models are generalizable across diverse patient populations is another critical challenge. External validation across multiple centers and continuous model updates are essential to ensure that AI systems perform well in real-world settings with varying patient demographics and imaging techniques. Lastly, regulatory approval from bodies like the FDA or EMA is necessary for clinical implementation of AI models, adding another layer of complexity to the integration process.

Overcoming these challenges is essential for unlocking the full potential of AI in revolutionizing cardiac CT diagnosis and improving patient care.

## Conclusion

Artificial intelligence (AI) is revolutionizing the field of cardiac CT, offering innovative solutions to improve diagnostic accuracy and clinical decision-making. Recent studies have demonstrated the remarkable potential of machine learning (ML) techniques, including radiomics and deep learning (DL), to predict the need for revascularization in coronary artery plaque segments. These methods, particularly when combined, have shown comparable performance to simulated FFR measurements, offering a faster and less invasive alternative for assessing the functional significance of coronary lesions.

**Fig. 3:**
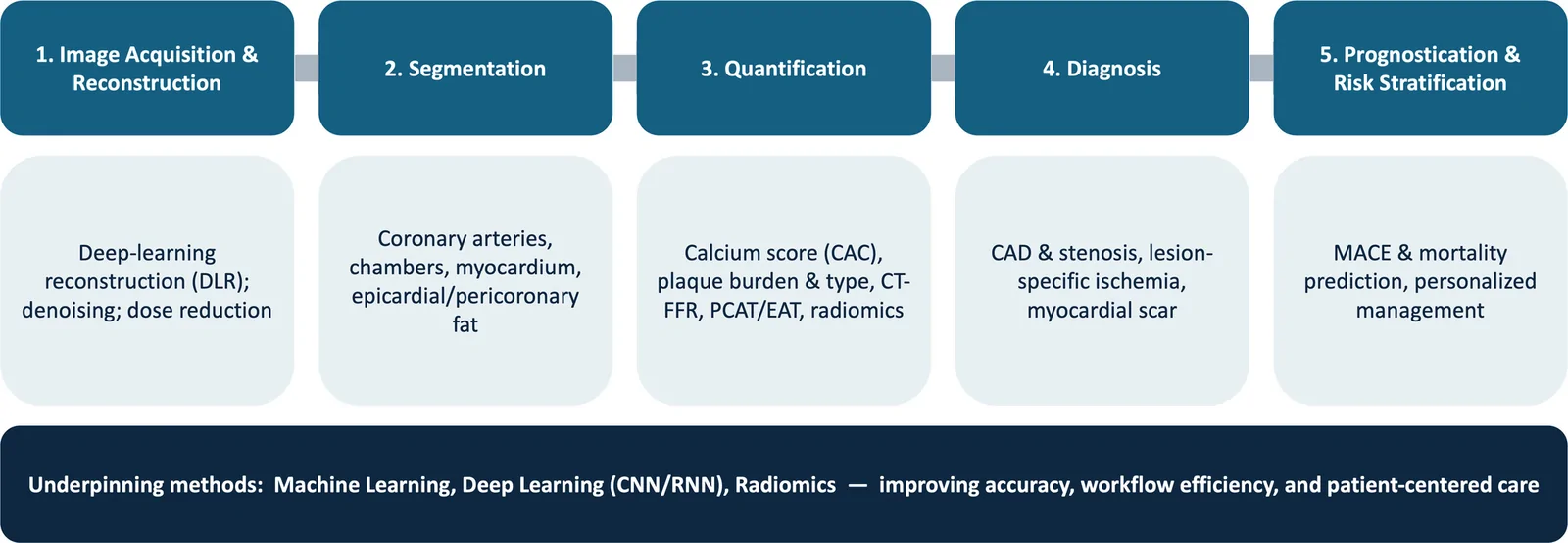
AI-enhanced cardiac CT workflow, showing the sequential stages in which AI contributes: image acquisition and reconstruction, segmentation, quantification (e.g., calcium score, plaque, CT-FFR), diagnosis, and prognostication for personalized management

As CCTA becomes the preferred method for ruling out stable coronary artery disease (CAD), the resulting increase in workload necessitates automation throughout the imaging process. AI is poised to play a pivotal role in streamlining data acquisition, post-processing, and interpretation, ultimately leading to a more efficient and precise approach to cardiovascular imaging (Fig. 3).

The applications of AI in cardiovascular CT are vast and varied, extending beyond image acquisition and reconstruction. AI algorithms can enhance image quality through denoising, accurately segment anatomical structures, perform quantitative analysis, provide diagnostic support, and even assist in decision-making. This integration of AI technologies not only improves diagnostic accuracy but also enables personalized treatment recommendations based on prognostic risk derived from both clinical data and imaging findings.

While the potential of AI in cardiac CT is undeniable, its successful implementation requires high-quality data and rigorous validation on diverse, unseen datasets. Continued advancements in AI algorithms and the standardization of imaging protocols will further improve accuracy and interpretability. As AI becomes increasingly integrated into standard cardiac image analysis software, healthcare professionals must develop proficiency in interpreting AI-generated results, understanding their limitations, and embracing promising applications to optimize patient care. In the near future, AI is likely to play a crucial role in clinical practice, ultimately leading to improved outcomes for patients with cardiovascular disease.

## Key References

- Griffin WF, Choi AD, Riess JS, et al. AI Evaluation of Stenosis on Coronary CTA, Comparison With Quantitative Coronary Angiography and Fractional Flow Reserve: A CREDENCE Trial Substudy. JACC Cardiovasc Imaging. 2023;16(2):193–205.

- *○ This CREDENCE substudy shows that AI-based stenosis evaluation on CCTA agrees closely with invasive quantitative coronary angiography and fractional flow reserve, validating AI as an accurate tool for non-invasive stenosis assessment.*

- Dundas J, Leipsic J, Fairbairn T, et al. Interaction of AI-Enabled Quantitative Coronary Plaque Volumes on Coronary CT Angiography, FFRCT, and Clinical Outcomes: A Retrospective Analysis of the ADVANCE Registry. Circ Cardiovasc Imaging. 2024;17(3):e016143.

- *○ This large registry analysis demonstrates that AI-enabled quantitative plaque volumes derived from CCTA, together with FFRCT, are associated with clinical outcomes, supporting the prognostic value of automated plaque quantification at scale.*

- Miller RJH, Shanbhag A, Killekar A, et al. AI-derived epicardial fat measurements improve cardiovascular risk prediction from myocardial perfusion imaging. Npj Digit Med. 2024;7(1):1–8.

- *○ This study shows that fully automated, AI-derived epicardial adipose tissue measurements add incremental value to cardiovascular risk prediction, highlighting AI's expanding role in imaging-based risk stratification.*

## Data Availability

No datasets were generated or analysed during the current study.
